# Modulation of Type III Secretion System in *Pseudomonas aeruginosa*: Involvement of the PA4857 Gene Product

**DOI:** 10.3389/fmicb.2016.00007

**Published:** 2016-01-28

**Authors:** Miao Zhu, Jingru Zhao, Huaping Kang, Weina Kong, Yuanyu Zhao, Min Wu, Haihua Liang

**Affiliations:** ^1^Key Laboratory of Resources Biology and Biotechnology in Western China, Ministry of Education, Department of Life Science, Northwest UniversityXi’an, China; ^2^Department of Biomedical Sciences, The University of North Dakota School of Medicine and Health SciencesGrand Forks, ND, USA

**Keywords:** type III secretion system, *tspR*, bacterial virulence, regulatory mechanisms, *Pseudomonas aeruginosa*

## Abstract

*Pseudomonas aeruginosa* is an opportunistic pathogen that causes serious acute or chronic infections in humans. Acute infections typically involve the type III secretion systems (T3SSs) and bacterial motility, whereas chronic infections are often associated with biofilm formation and the type VI secretion system. To identify new genes required for pathogenesis, a transposon mutagenesis library was constructed and the gene PA4857, named *tspR*, was found to modulate T3SS gene expression. Deletion of *P. aeruginosa tspR* reduced the virulence in a mouse acute lung infection model and diminished cytotoxicity. Suppression of T3SS gene expression in the *tspR* mutant resulted from compromised translation of the T3SS master regulator ExsA. TspR negatively regulated two small RNAs, RsmY and RsmZ, which control RsmA. Our data demonstrated that defects in T3SS expression and biofilm formation in *retS* mutant could be partially restored by overexpression of *tspR*. Taken together, our results demonstrated that the newly identified *retS-tspR* pathway is coordinated with the *retS-gacS* system, which regulates the genes associated with acute and chronic infections and controls the lifestyle choice of *P. aeruginosa*.

## Introduction

*Pseudomonas aeruginosa* is one of the most common nosocomial pathogens and often causes numerous acute or chronic infections (Deretic et al., 1995). The acute infections mainly rely on the expression of specific virulence factors, such as flagella, pili, exotoxin, and the type III secretion system (T3SS) (Sadikot et al., 2005). Chronic *P. aeruginosa* infections, such as pulmonary infections in cystic fibrosis (Brencic et al., 2009), are often accompanied by the formation of bacterial biofilm communities, which is the major barrier to eradicate *P. aeruginosa* chronic infections (Deretic et al., 1995; Parsek and Singh, 2003; Morita et al., 2014).

The ability of *P. aeruginosa* to avoid phagocytic clearance is a major virulence determinant that primarily depends on the T3SS (Dacheux et al., 1999; Rangel et al., 2014). *P. aeruginosa* uses its T3SS to produce and directly inject four virulence effectors (ExoS, ExoT, ExoY, and ExoU; Frank, 1997; Ghosh, 2004; Sato and Frank, 2011) in addition to nucleoside diphosphate kinase (NDK; Neeld et al., 2014) into host cells that disrupt intracellular signaling or cell death (Francis et al., 2002). The T3SS of *P. aeruginosa* has been shown to contribute to epithelial cell and macrophage damage *in vitro*, in animal models of disease, and in human infections (Roy-Burman et al., 2001; Smith et al., 2004; Engel and Balachandran, 2009).

Type III secretion system gene expression is activated by numerous environmental signals including bacterial contact with host cells and growth in low calcium conditions (Frank, 1997; Hueck, 1998; Vallis et al., 1999). The regulation of T3SS is achieved through a complex regulatory network (Yahr and Wolfgang, 2006; Diaz et al., 2011) controlled by a master regulator ExsA, which is an AraC-family that recognizes and binds to a consensus sequence located upstream of the transcriptional start sites of T3SS genes (Hovey and Frank, 1995). The two-component system AlgR/FimS recalibrates the RsmAYZ post-transcriptional regulatory system to suppress the T3SS in the context of a mucoid background (Wu et al., 2004; Jones et al., 2010; Intile et al., 2014). Alteration of intracellular cAMP levels also affects T3SS expression, and cAMP influences gene expression by acting as an allosteric regulator of Vfr, which is required for the expression of quorum-sensing, exotoxin A production and Type IV pilus-mediated twitching motility genes (West et al., 1994; Albus et al., 1997; Beatson et al., 2002). Microarray analyses revealed that *vfr* mutation reduced T3SS expression (Wolfgang et al., 2003). Overexpression of PtrA or PtrB, which are induced by copper stress or the SOS response, represses T3SS (Ha et al., 2004; Wu and Jin, 2005; Elsen et al., 2011). Moreover, deletion of genes related to metabolic processes also leads to inhibition of T3SS expression (Rietsch et al., 2004; Linares et al., 2010). These findings indicate that the T3SS of *P. aeruginosa* is tuned by various environmental stresses, which might be an important survival strategy for this microorganism.

In addition to environmental stresses, other signaling pathways also control the T3SS. The development of acute versus chronic infection has been shown to be controlled by the hybrid sensor kinases LadS and RetS, although the cognate signals are unknown. *retS* mutation reduced T3SS expression and increased biofilm formation (Goodman et al., 2004; Laskowski et al., 2004). In contrast, LadS had a negative impact on T3SS gene expression but a positive effect on biofilm formation (Ventre et al., 2006). Thus, RetS and LadS are thought to act reciprocally to control the switch between acute and chronic infection (Ventre et al., 2006; Goodman et al., 2009). Moreover, both RetS and LadS interact with another two-component system GacS/GacA, in which GacS is the sensor kinase and GacA is the response regulator. RetS inhibits GacS by forming a RetS/GacS heterodimer and, blocking phosphor transfer from GacS to GacA, however, how LadS interacts with GacS/GacA is not yet known (Goodman et al., 2009). GacS/GacA positively regulates the expression of two small RNAs (sRNAs), RsmY and RsmZ (Kay et al., 2006; Brencic et al., 2009), which are antagonists of the RNA binding regulator RsmA. Upregulation of RsmY and RsmZ leads to T3SS inhibition and a hyperbiofilm phenotype (Brencic et al., 2009; Bordi et al., 2010). RsmA is a global post-transcriptional regulatory protein, which controls the switch between T3SS activation and biofilm formation (Mulcahy et al., 2006; Irie et al., 2010; Kulkarni et al., 2014) and indirectly controls T3SS in mucoid *P. aeruginosa* (Intile et al., 2014). Although the functions of the T3SS in *P. aeruginosa* have been widely studied, the regulatory mechanisms still remain elusive. In this study, we identified *tspR* as an essential gene for T3SS expression in *P. aeruginosa*. *In vivo* studies indicate that TspR plays an important role in *P. aeruginosa* pathogenesis. TspR influences the expression of the master T3SS regulator ExsA at transcriptional and post-transcriptional level. Our studies demonstrated that *tspR* and *retS* mutants have similar phenotypes, such as inhibition of T3SS activity and induction of hyperbiofilm formation. In addition, TspR negatively controls two sRNAs, RsmY and RsmZ, and consequently the T3SS. This study reports a new gene involved in the T3SS regulatory network that controls acute and chronic *P. aeruginosa* infections.

## Materials and Methods

### Strains, Plasmids, and Growth Conditions

The bacterial strains and plasmids used in this study are listed in **Supplementary Table S1**. *P. aeruginosa* PAO1 and derivatives were grown at 37°C on LB agar plates or in LB broth with shaking at 220 rpm unless otherwise specified. LB was used as a T3SS non-inducing medium and LB supplemented with 5 mM EGTA and 20 mM MgCl_2_ as a T3SS inducing medium (calcium-deplete). Antibiotics were used at the following concentrations: for *Escherichia coli*, gentamicin (Gm) at 15 μg/ml, ampicillin at 100 μg/ml, and tetracycline 10 μg/ml; for *P. aeruginosa*, gentamicin at 50 μg/ml in LB or 150 μg/ml in PIA (*Pseudomonas* Isolate Agar); tetracycline at 150 μg/ml in LB or 300 μg/ml in PIA and carbenicillin at 500 μg/ml in LB.

### Transposon Mutagenesis Library

The transposon mutagenesis library was constructed as previously described except some modifications (Kulasekara et al., 2005). Briefly, the donor strain (*E. coli* SM10) containing pBT20, and the recipient strain PAO1(*CTX-exoS-lux*; Liang et al., 2014) were scraped from overnight-incubated plates before the cells were collected. The cells were resuspended in LB and spotted on a fresh LB agar plate at a ratio of 2:1. After incubation for 2 h, the mixed culture was diluted and spread on PIA (*Pseudomonas* isolation agar) plates containing Gm at 150 μg/ml. A transposon mutant library was constructed by picking 8,000 colonies grown on the selective plates (PIA + 150 μg/ml Gm). After overnight incubation, colonies with changed activities of CTX-*exoS-lux* under a Tannon imaging system (Tannon 5500) were collected.

To further screen for genes tuning *exoS-lux* expression, the random mutant library was cultured overnight in LB medium supplemented with Gm at 50 μg/ml. Three additional re-screens were performed to eliminate false-positive clones. During re-screening, the overnight cultures were diluted 1:300 in the appropriate media in 96-well clear-bottom black plates (Costar 9520, Corning) and were assayed for both luminescence and absorbance over the experimental time course. The transposon insertion sites of the selected mutants were determined by an arbitrary primed polymerase chain reaction (PCR) and subsequent sequencing of the PCR products.

### Construction of Plasmids

To construct the *p-tspR* and *p-retS* plasmids, the fragments of *tspR* and *retS* were respectively amplified by PCR with the corresponding primer pairs: Com-*tspR*-S/Com-*tspR*-A and Com-*retS*-S/Com-*retS*-A. The PCR products were digested with the indicated enzymes and cloned into PAK1900 (Wolfe, 2005).

The plasmid pMS402 carrying a promoter-less *luxCDABE* reporter gene cluster was used to construct promoter-*luxCDABE* reporter fusions as described previously (Duan et al., 2003; Liang et al., 2011). For generating *tspR-lux*, the *tspR* promoter region (-522 to +77 from *tspR* translational starting site) was amplified by PCR using the primers *tspR-lux*-S (with *Xho*I site) and *tspR-lux*-A (with *Bam*HI site). The PCR products were cloned into the pMS402, yielding P*tspR-lux*. Besides the plasmid-based reporter system, an integration plasmid CTX6.1 originating from plasmid mini-CTX-*lux* was used to construct chromosomal fusion reporter. The pMS402 fragment containing the kanamycin-resistance marker, the multiple cloning sites (MCSs), and the promoter-*luxCDABE* reporter cassette was then isolated and ligated into CTX6.1, yielding *tspR-lux*. The plasmid generated was first transferred into *E. coli* SM10-λ *pir* and the *P. aeruginosa* reporter integration strain was obtained using bi-parental mating as previously reported (Hoang et al., 2000). The same procedures were used for generating other promoter-*lux* fusions except the different primers (**Supplementary Table S2**). All constructs were sequenced to verify that no mutations occurred in these constructs.

### Luminescence Screening Assays

The expression of the *lux*-based reporters from bacteria grown in liquid culture was measured as counts per second of light production using a Synergy 2 Plate Reader (BioTek) as previously described (Liang et al., 2008). Overnight cultures of the reporter strains were diluted to an OD_600_ of 0.2 and shaken for an additional 2 h before use. The cultures were inoculated into parallel wells of a black 96-well plate with a transparent bottom. A 5-μl volume of the fresh culture was inoculated into the wells containing a total volume of 95-μl mediums, which made the OD_600_ around 0.07. A 60-μl volume of filter-sterilized mineral oil was added to each well to prevent evaporation during the assay. Promoter activities were measured every 30 min for a 24 h course. Bacterial growth was monitored at the same time by measuring the OD at 595 nm.

### Construction of *P. aeruginosa tspR*::Gm, *exsA*::Gm*, retS*::Gm*, rsmY*::Gm*, rsmZ*::Gm*, rsmY*::Gm*/tspR*::Tc, *rsmZ*::Gm*/tspR*::Tc Mutants

For construction of gene knockout mutants, a SacB-based strategy was employed as previously described (Hoang et al., 1998; Liang et al., 2014). To construct the *tspR* null mutant (Δ*tspR*), PCRs were performed to amplify sequences upstream (1959 bp) and downstream (1463 bp) of the intended deletion. The upstream fragment was amplified from PAO1 genomic DNA using primer pair, pEX-*tspR*-up-S and pEX-*tspR*-up-A, while the downstream fragment was amplified with primer pair, pEX-*tspR*-down-S and pEX-*tspR*-down-A (**Supplementary Table S2**). The two PCR products were digested and then cloned into *Bam*HI/*Hin*dIII-digested gene replacement vector pEX18Ap, yielding pEX18Ap-*tspR*. A 0.9 kb gentamicin resistance cassette cut from pPS858 with *Xba*I was cloned into pEX18Ap-*tspR*, yielding pEX18Ap-*tspR*-Gm. The resultant plasmids were electroporated into PAO1 with selection for gentamicin resistance. Colonies showing both gentamicin resistance and loss of sucrose (5%) susceptibility were selected on LB agar plates containing 50 μg/ml of gentamicin and 5% sucrose, which typically indicates a double-crossover event and thus of gene replacement occurring. The pEX18Ap-*tspR*-Tc was constructed by a similar strategy as described above. A 2.3 kb tetracycline resistance cassette was amplified from integration vector mini-CTX-*lacZ* with primer pair Tc-S/Tc-A (with *Xba*I site; **Supplementary Table S2**) for replacing the *tspR* gene in PAO1. The *tspR*::Tc mutant was further confirmed by PCR. The *exsA*::Gm, *retS*::Gm, *rsmY*::Gm and *rsmZ*::Gm mutants (contains the Gm marker) were generated by a similar strategy with deletion of the *tspR* gene in PAO1.

For generating *tspR/rsmY, tspR/rsmZ* double mutants (*rsmY*::Gm*/tspR*::Tc, *rsmZ*::Gm*/tspR*::Tc), the *tspR* gene in Δ*rsmY*, Δ*rsmZ* mutant was deleted by a similar strategy with plasmid pEX18Ap-*tspR*-Tc. These resultant mutants were verified by PCR.

### Cytotoxicity Assay

Bacterial cytotoxicity was determined by measuring the detachment of the A549 cells after *P. aeruginosa* infection as previously described (Wu and Jin, 2005; Li et al., 2013) with minor modifications. The human A549 lung epithelial cells (6 × 10^5^) were seeded into each well of a 12-well plate. The cells were cultured in RPMI medium supplemented with 10% fetal bovine serum at 37°C with 5% CO_2_ for 24 h. Overnight bacterial culture was subcultured in fresh LB to the log phase before infection. Subsequently, bacterial were washed three times with phosphate-buffered saline (PBS) and resuspended in PBS. A549 cells were then infected with the bacteria at a multiplicity of infection (MOI) of 20. After 4 h infection, the culture medium in each well was aspirated. Cells were washed three times with PBS and stained with 200 μl 0.1% crystal violet (CV)-10% ethanol for 15 min at 37°C. The staining solution was discarded, and the plates were washed twice with 1 ml water. A-250 μl volume of 95% ethanol was then added into each well, and the reaction mixture was incubated at room temperature for 30 min with gentle shaking. The ethanol solution with dissolved CV dye was subjected to measurement of absorbance at a wavelength of 590 nM. To observe the morphology phenotype of A549 cells infected after *P. aeruginosa*, the cells fixed by 4% paraformaldehyde for 15 min, washed three times with PBS, and dyed by Giemsa stain for about 12 min. Cells were observed under microscope.

### Western Blot Analysis

Overnight cultures of the tested strains were transformed into the same fresh LB medium or with 5 mM EGTA and 20 mM MgCl_2_ to an A_600_ of 0.02 and cultivated for additional 3 h. 100-μl cultures were centrifuged and the pellets were resuspended in 10 μl PBS. Bacterial cells were loaded and separated by 12% SDS-PAGE. The proteins were transferred onto a PVDF membrane and hybridized with a rabbit polyclonal ExoS antibody (1: 2000 dilutions, from Shouguang Jin and Weihui Wu’s Laboratory) or a mouse monoclonal FLAG antibody (Sigma). The signal was detected by an ECL Plus kit (Amersham Biosciences).

### Biofilm Formation Assay

Biofilm formation was measured in a static system as previously described (O’Toole and Kolter, 1998) with minor modifications. Visualization of biofilm formation was carried out in 15-mL borosilicate tubes. Briefly, Cells from overnight cultures were inoculated at 1:100 dilutions into LB medium supplemented with appropriate antibiotics and grown at 30°C for 8 h. Biofilms were stained with 0.1% CV and tubes were washed with water to remove unbound dye.

Quantification of biofilm formation was performed in 24-well polystyrene microtiter plates. LB and appropriate antibiotics was inoculated to a final OD_600_ of 0.01. The plates were incubated for 8 or 20 h at 30°C. CV was added to each tube and stained for 15 min prior to removal by aspiration. Wells were rinsed three times by submerging the tubes in distilled water, and the remaining CV was dissolved in 1 ml of 95% ethanol. A 1 ml portion of this solution was transferred to a new polystyrene tube, and the absorbance was measured at 600 nm.

### Swarming Motility Assay

Bacterial swarming motility was assessed as described previously (Rashid and Kornberg, 2000) with slight modification. The medium used for the swarming motility assay consisted of 0.5% agar, 8 g/L nutrient broth mix, and 5 g/L glucose. Bacteria were spot inoculated onto plates as 2 μl aliquots taken directly from overnight LB cultures. The plates were incubated at 37°C for 12 h. Photographs were taken with the Tanon 2500 imaging system.

### Murine Acute Pneumonia Model

Bacteria were grown overnight in LB broth at 37°C with shaking at 200 rpm. The next day, the bacteria were pelleted by centrifugation at 5,000 × *g* and suspended in 10 ml of fresh LB broth and allowed to grow until the mid-logarithmic phase. OD_600__nm_ was measured; density was adjusted to OD ≈0.25. C57BL6 mice were purchased from the Harlan Laboratory (Indianapolis, IN, USA). The animal experiments have been approved by the University of North Dakota institutional animal care and use committee (UND IACUC Approval #1204-4). Mice were randomly assigned to different groups (six each group), and were lightly anesthetized with 20 mg/kg ketamine plus 5 mg/kg diazepam. Then we intranasally instilled 5 × 10^7^ colony-forming units (CFUs) of *P. aeruginosa* and monitored the animals with infection for up to 120 h. Intranasal instillation of equal amount of PBS was performed as controls. Moribund mice were euthanized to obtain the lung for analysis. Survival of the mice was monitored for 5 days after the infection.

## Results

### Identification of *P. aeruginosa* Mutants with Altered *exoS* Expression

*Pseudomonas aeruginosa* utilizes the T3SS to translocate four effectors, ExoS, ExoY, ExoT and ExoU, into the cytoplasm of host cells. Changes in the expression of the T3SS often alter disease progression. To investigate the regulatory mechanism of the T3SS, we constructed a transposon insertion library in a wild-type PAO1 strain containing an *exoS-lux* transcriptional reporter integrated into the chromosome at the vacant phage CTX attachment site and screened for mutants with altered *exoS-lux* expression. Fifteen mutants were selected and the insertion sites were determined by arbitrary primed PCR and DNA sequencing. The mutated genes are listed in **Table 1**.

**Table 1:** List of mutants showing more than threefold changes in the *exoS-lux* expression.

Gene name or number	Insertion site	Protein description	Max fold^b^
*PA0716/PA0717*	788912	Hypothetical protein	3.4
*PA2621* (*clpS*)	2964732	ATP-dependent Clp protease adaptor	7.2
*PA3284*	3676853	Hypothetical protein	6.0
*PA0265* (*gabD*)	300415	Succinate-semialdehyde dehydrogenase	-6.5
*PA1056* (*shaC*)	1146026	Proton transport	-4.0
*PA1703* (*pcrD*)	1850681	Type III secretory apparatus protein	-7.0
*PA1713* (*exsA*)	1857602	Transcriptional regulator ExsA	NE^a^
*PA1716* (*pscC*)	1860621	Type III secretion outer membrane protein PscC precursor	-7.5
*PA2227* (*vqsM*)	2449253	AraC-type transcriptional regulator	-5.6
*PA2550*	2882043	Probable acyl-CoA dehydrogenase	-5.8
*PA2647* (*nuoL*)	2993165	NADH dehydrogenase I chain L	-3.5
*PA2840* (*deaD*)	3194769	Probable ATP-dependent RNA helicase	-10
*PA3115* (*fimV*)	3498191	Motility protein FimV	-11
*PA4857* (*tspR*)	5455890	Hypothetical protein	-4.5
*PA5264*	5926218	Hypothetical protein	-4.7


*^a^NE, No expression of *exoS* was observed in the mutant.*

*^b^Max fold, Maximal ratio of expression between the mutant and the wild-type.*

Among these 15 genes, transposon insertion into three genes caused increased expression of *exoS*, and insertion into 12 genes caused decreased expression. As expected, a group of known T3SS modulators including PcrD, PscC and ExsA, were also isolated (Yahr et al., 1997). *clpS* encodes an ATP-dependent Clp protease adaptor that is associated with antibiotic resistance, motility, and biofilm formation of *P. aeruginosa* (Fernandez et al., 2012). ClpS may interact with a protein (s) that controls T3SS expression and is degraded by ClpAP protease. Consistent with our findings, the AraC-family transcriptional regulator VqsM directly binds to the promoter region of the master regulator ExsA, and regulates T3SS expression (Liang et al., 2014). PA0265 (*gabD*) and PA2550 are involved in amino acid metabolism, while NuoL and ShaC are associated with energy metabolism. These results are consistent with previous studies showing that metabolic imbalance leads to T3SS inhibition (Dacheux et al., 2002; Rietsch et al., 2004). DeaD is an RNA helicase that stimulates ExsA translation to promote expression of the T3SS (Intile et al., 2015). We also observed that disruption of *fimV* in *P. aeruginosa* reduced *exoS* activity. FimV positively regulates twitching and type II secretion system when grown on solid medium as well as intracellular cAMP levels (Semmler et al., 2000; Fulcher et al., 2010; Michel et al., 2011). FimV may regulate T3SS expression by controlling intracellular cAMP concentrations. Finally, PA0716, PA3284, PA4857, and PA5264 encode hypothetical proteins, whose detailed effects on T3SS regulation need to be further investigated.

### TspR is Required for T3SS Expression, Biofilm Formation, and Bacterial Motility

**FIGURE 1:**
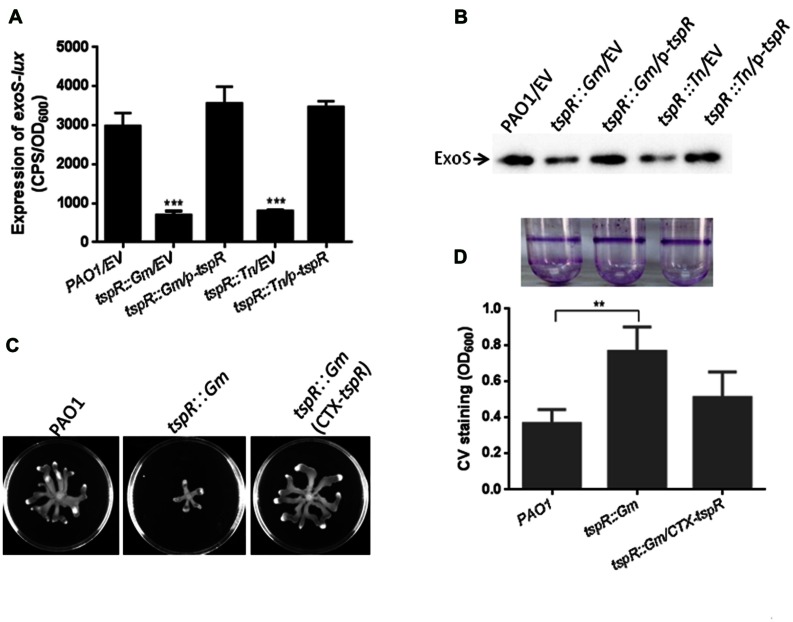
**TspR is required for the expression of T3SS genes.** **(A)** The expression of *exoS-lux* was measured in the wild-type PAO1, *tspR*::Gm, and the complemented *tspR*::Gm strain (*tspR*::Gm/p-*tspR*), the *tspR* transposon strain (*tspR*::Tn), and the *tspR*::Tn complemented strain (*tspR*::Tn/p-*tspR*). The asterisks indicate the statistically different *exoS* expression compared to that in the wild-type strain as determined by Student’s *t*-test (^∗∗∗^*P* < 0.001). **(B)** The expression and secretion of ExoS were examined in the indicated strains by Western-blotting. Strains were grown in LB with EGTA to an OD_600_ = 0.6. Whole-cell extracts from the designated strains were separated by SDS-PAGE and immuno-blotted. **(C)** Effect of *tspR* mutation on swarming motility. Overnight cultures were spotted onto swarming plates (2 μl aliquots) and the plates were incubated at 37°C. The images captured after 16 h of growth. The experiments were repeated at least three times and similar results were observed. **(D)** TspR is required for biofilm formation. Quantification of crystal violet (CV) staining of biofilms was grown in microtiter plates for 14 h. ^∗∗^*P* < 0.005 based on Student’s *t*-test. Photo of the tube binding assay was taken. EV, empty vector.

Among the 15 genes that regulate T3SS, we investigated the role of PA4857, which we designated as Type III secretion system and pathogenesis Regulator (*tspR*), in T3SS regulation. *tspR* is located immediately downstream of *retS*, a known regulator of T3SS gene expression and biofilm formation in *P. aeruginosa* (Goodman et al., 2004; Laskowski et al., 2004). To verify if *tspR* mutation decreased *exoS* expression, we generated a chromosomal *tspR* mutation (*tspR*::Gm) in PAO1 strain, and measured *exoS* promoter activity in this mutated strain. Expectedly, the levels of *exoS* transcription were reduced by about threefold in the *tspR* mutated strain compared to the levels in the wild-type strain, which is consistent with observation in the original *tspR* transposon mutant (**Figure 1A**). Introduction of a plasmid expressing TspR (p-*tspR*) into the *tspR*::Gm strain restored *exoS-lux* activity to wild-type levels (**Figure 1A**). To further confirm the role of TspR in the regulation of *exoS* expression, Western-blotting was used to measure the levels of ExoS protein in the wild-type PAO1, *tspR*::Gm, and transposon mutant (*tspR*::Tn) strains, as well as their corresponding complemented strains (*tspR*::Gm/p-*tspR*, and *tspR*::Tn/p-*tspR*). As shown in **Figure 1B**, lower levels of ExoS were detected in the *tspR*::Gm and *tspR*::Tn mutant strains than in the wild-type strain. In the complemented strains, ExoS expression was restored to wild-type levels. These results clearly indicate that TspR is a positive modulator of T3SS expression.

To define the function of TspR, its effects on biofilm formation and bacterial motility were also examined. Mutation of *tspR* resulted in reduced swarming motility (**Figure 1C**) and increased biofilm formation, as evidenced by the results of static CV and tube binding assays (**Figure 1D**). These phenotypes are consistent with those previously reported in the *retS* mutant (Goodman et al., 2004), suggesting that *tspR* and *retS* may be involved in the same regulatory pathway.

### Deletion of *tspR* Diminishes Cytotoxicity and Reduces Virulence in a Mouse Model of Acute Pneumonia

**FIGURE 2:**
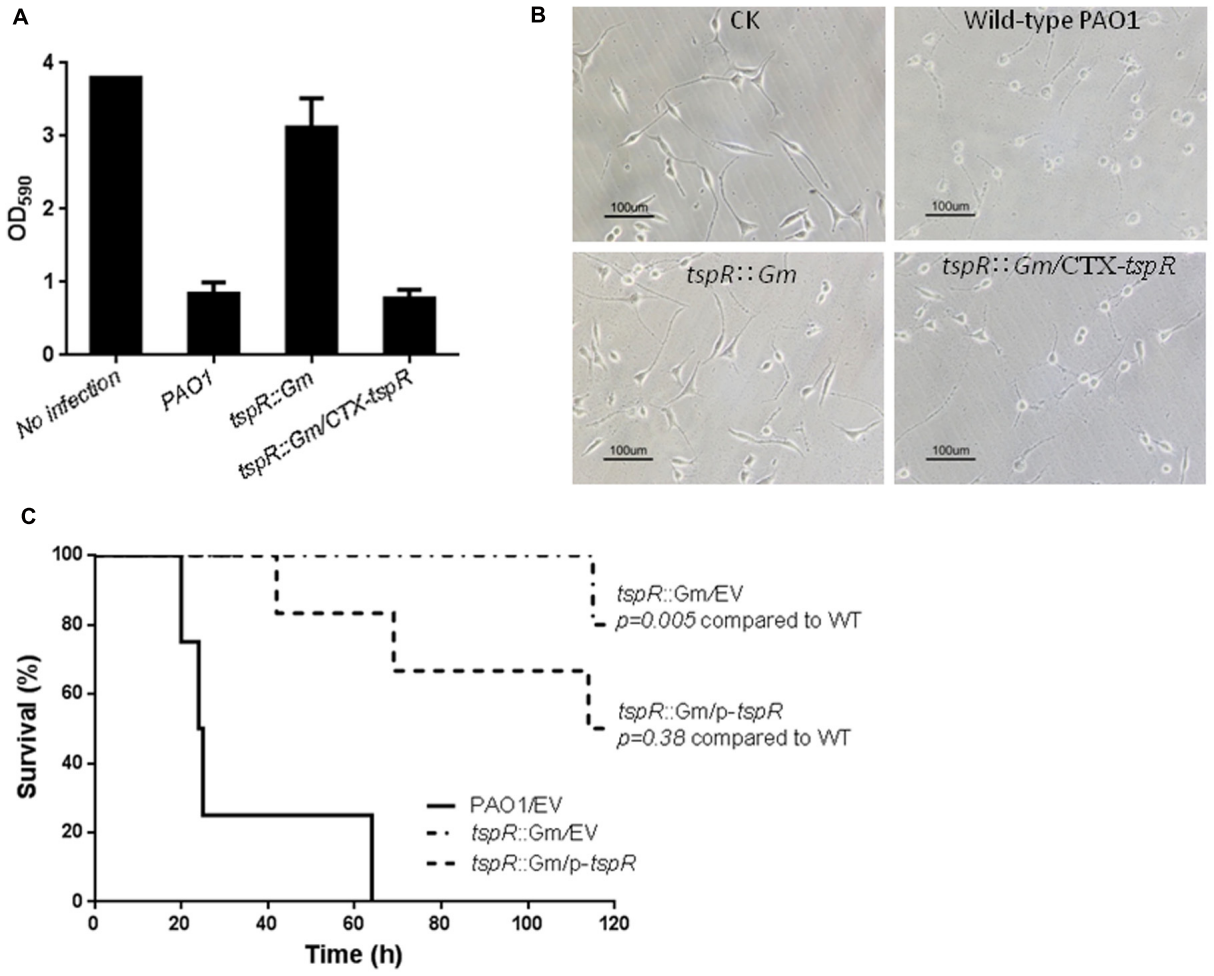
**Mutation of *tspR* diminishes cytotoxicity and reduced the virulence of *P. aeruginosa*.** **(A)** Cytotoxicity of the *P. aeruginosa* and its derivates. A549 cells were infected with the indicated strains at a MOI of 20. At 4 h post-infection, cells attached to the plate were measured with CV staining. Results represent mean ± SD, and data are representative of three independent experiments. **(B)** Microscopy images of A549 cells infected with the indicated strains. The control (CK) was an uninfected A549 culture. **(C)** The *tspR* mutation reduced the virulence of *P. aeruginosa*. C57BL6 mice were intranasally challenged with the wild-type strain, the *tspR*::Gm strain, and the complemented *tspR*::Gm strain at 5 × 10^7^ CFU in 50 μl of PBS, and moribund mice were killed to determine survival (Kaplan–Meier Curve with Log-Rank test, *P* = 0.0028, *n* = 6).

Since deletion of *tspR* significantly compromised the expression of T3SS genes, and bacterial motility (**Figure 1**), T3SS-mediated cytotoxicity was examined by measuring cells remaining attached after infection. A549 cells were infected with the wild-type PAO1, the *tspR*::Gm strain, or the complemented strain (*tspR*::Gm/p-*tspR*) at a MOI of 20. After 4 h post-infection, the majority of the cells were rounded and detached. As shown in **Figure 2A**, mutation of *tspR* rendered less cytotoxicity than the wild-type strain, and complementation with a *tspR* gene restored the cytotoxicity. Moreover, few living A549 cells could be observed under microscope 4 h after infection with the wild-type PAO1 strain, while most A549 cells remained alive after infected with the *tspR*::Gm strain (**Figure 2B**). These results demonstrated that the loss of *tspR* attenuates the cytotoxicity of *P. aeruginosa*, further verifying its impact on T3SS.

Chronic *P. aeruginosa* lung infection is a major cause of morbidity and mortality in cystic fibrosis patients. To further investigate the importance of TspR in pulmonary infection, C57BL/6 mice were infected intranasally with approximately 5 × 10^7^ cells of the wild-type, *tspR*::Gm or complemented (*tspR*::Gm/p-*tspR*) strains. Kaplan–Meier survival analysis showed that mutation of *tspR* significantly improved mouse survival compared to that of mice infected with the wild-type strain. Mice infected with the *tspR*::Gm strain exhibited significantly decreased mortality with no death until 115 h post-infection and more than 80% of mice surviving at 120 h. In contrast, wild-type PAO1 infection resulted in 50 and 100% mortality at 24 and 60 h post-inoculation, respectively (**Figure 2C**). Complementation of the deletion strain (*tspR*::Gm/p-*tspR*) partially restored the lethal infection phenotype to the wild-type level. Taken together, these results clearly demonstrated that TspR is essential for the virulence of *P. aeruginosa* in a mouse model of acute infection.

### TspR Controls the Expression of ExsA at the Transcriptional and Post-transcriptional Level

**FIGURE 3:**
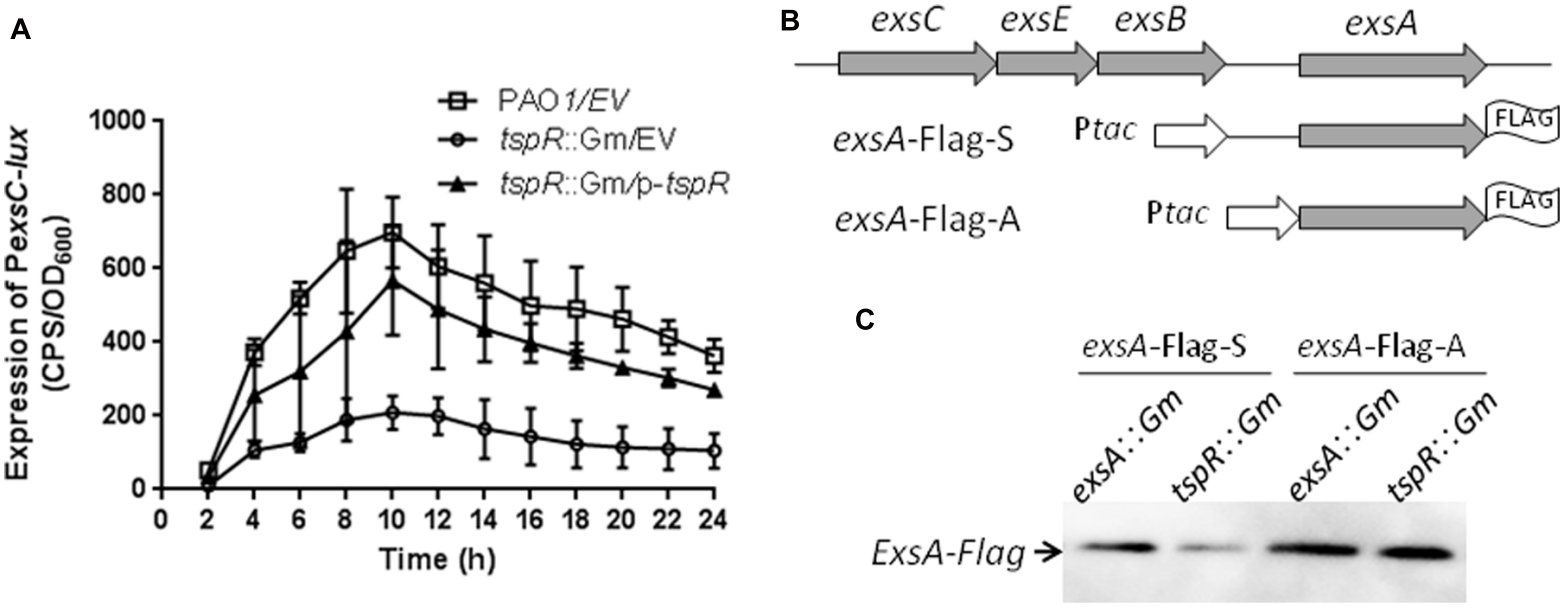
**TspR affects expression of the *exsCEBA* operon at the transcriptional and post-transcriptional levels.** **(A)** The activity of P*exsC-lux* was determined in the wild-type and the *tspR*::Gm strains. The results shown are the average of triplicate experiments. Error bars indicate the standard deviations. EV, empty vector. **(B)** *exsA*-FLAGs constructs. P*tac* represents a *tac* promoter. **(C)** Western-blotting showing the expression levels of ExsA-FLAG under T3SS-inducing conditions in the *exsA* and *tspR* mutants.

Expression of the T3SS genes is finely tuned by multiple environmental conditions. The two best known signals for T3SS gene expression are contact with host cells and extracellular calcium (Ca^2+^) concentrations in the micromolar range (Frank, 1997; Vallis et al., 1999). In addition, T3SS expression is controlled by a complex regulatory network. Previous studies have shown that all T3SS genes are under the direct transcriptional control of ExsA, an AraC-family regulator (Hovey and Frank, 1995). Given that the expression of T3SS was also controlled by *tspR*, we hypothesized that TspR is epistatic of ExsA and regulates T3SS gene expression. *exsA* is co-transcribed with *exsC*, *exsE*, and *exsB* in the same operon, which is driven by a promoter upstream of *exsC*. Moreover, the expression of *exsC* is also controlled by ExsA (Yahr and Frank, 1994). Thus, we measured the expression of the *exsC-lux* fusion P*exsC-lux* in the wild-type and *tspR*::Gm strains. As shown in **Figure 3A**, expression of P*exsC-lux* was reduced in the *tspR*::Gm strain compared to that in the wild-type strain, and complementation with the *tspR* gene partially restored *exsC* promoter activity (**Figure 3A**), which indicates that TspR is involved in the regulation of *exsCEBA* operon expression.

We also observed that the decreased expression of *exsC* in the *tspR*::Gm strain was not due to a reduced *exsA* mRNA or protein level (data not shown). Thus, we attempted to test whether the inhibition of *exsCEBA* transcription observed in the *tspR* mutant might be due to reduced *exsA* expression at the post-transcriptional level. To this end, we transformed two *exsA*-FLAG fusions with different *exsA* upstream regions, *exsA*-FLAG-S and *exsA*-FLAG-A (**Figure 3B**; Li et al., 2013), into an *exsA* mutant or the *tspR* mutant. Expression of ExsA-FLAG was then examined under T3SS-inducing conditions. As shown in **Figure 3C**, ExsA-FLAG protein levels (from the *exsA*-Flag-A construct) in the *exsA*::Gm and *tspR*::Gm strains were similar, suggesting that P*tac* activity is not affected by the *tspR* mutation. However, when the *exsA* endogenous ribosome binding site and neighboring regions were included in the construct (*exsA*-Flag-S), the expression levels of ExsA-FLAG were reduced in the *tspR* mutant compared to those in the *exsA* mutant (**Figure 3C**). These results indicated that TspR controls the expression of ExsA at the post-transcriptional level.

### RetS is Required for the *tspR* Expression

**FIGURE 4:**
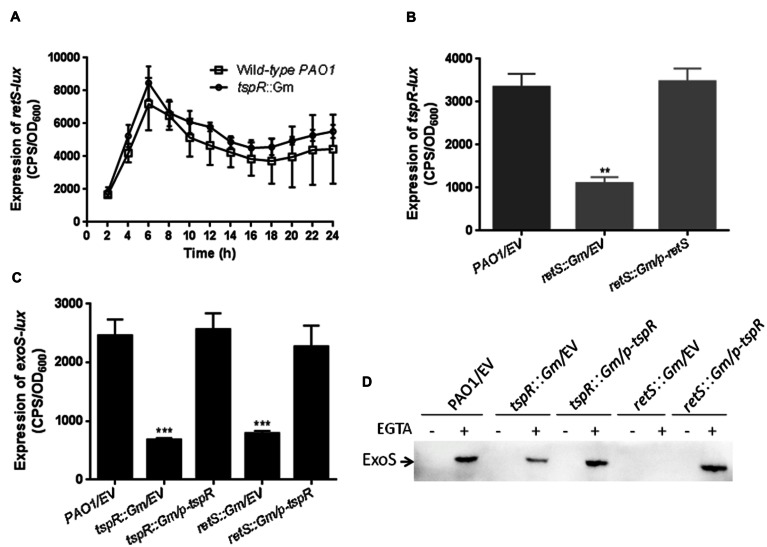
**RetS tunes the expression of *tspR*.** **(A)** TspR had no effect on *retS* expression. The level of *retS* transcription was measured in the wild-type and *tspR*::Gm strains. The results shown are the mean ± SD, and data are representative from three independent experiments. **(B)** RetS positively regulates the activity of *tspR*. The activity of the *tspR* promoter-*lux* fusion (*tspR-lux*) in the wild-type, *retS*::Gm and *retS*::Gm complemented strains were measured. ^∗∗^*P* < 0.005 between the wild-type and *retS* mutant by Student’s *t*-test. **(C)** The expression of *exoS* in the indicated strains (^∗∗∗^*P* < 0.001). **(D)** Overexpression of *tspR* in the *retS*::Gm mutant restored the expression of ExoS to wild-type levels. Indicated strains were grown in LB with or without EGTA to an OD_600_ = 0.6. Whole-cell extracts from the designated strains were separated by SDS-PAGE separation and subjected to Western-blotting. EV represents empty vector.

In addition to ExsA, the RetS and LadS sensor proteins also regulate T3SS expression in *P. aeruginosa*. Deletion of *retS* compromised T3SS genes expression and T3SS-dependent host cell cytotoxicity, but increased biofilm formation (Goodman et al., 2004; Laskowski et al., 2004). Interestingly, the *tspR* gene is located next to *retS* on the chromosome, which led us to investigate the possible interaction between *tspR* and *retS*. To this end, we constructed a *retS* promoter-*lux* fusion (*retS-lux*) and measured *retS-lux* activity in the wild-type and *tspR*::Gm strains. As shown in **Figure 4A**, the expression of *retS-lux* in the *tspR*::Gm strain was the same as that in the wild-type strain. We then examined the impact of RetS on *tspR-lux* expression. The activity of *tspR-lux* was about threefold lower in the *retS*::Gm strain than in the wild-type (**Figure 4B**), indicating that *retS* is a positive regulator of *tspR* expression.

Given that *retS* positively regulates the expression of both T3SS genes and *tspR*, we next examined whether the reduced *exoS-lux* activity observed in the *retS*::Gm strain could be restored by the expression of *tspR*. To this end, *exoS-lux* activity was measured in the wild-type, *tspR*::Gm, and *tspR*::Gm complemented (*tspR*::Gm/p-*tspR*) strains, as well as the *retS*::Gm mutant, and the *retS*::Gm strain carrying a p-*tspR* plasmid. As expected, the decreased *exoS* level in the Δ*retS* strain could be restored by *tspR* overexpression (**Figure 4C**). This observation was also confirmed by the Western-blot analysis with an antibody against ExoS (**Figure 4D**). In summary, these results indicated that *tspR* partially controls the RetS-mediated regulatory pathways.

### TspR Functions through Two Small RNAs RsmY an RsmZ

**FIGURE 5:**
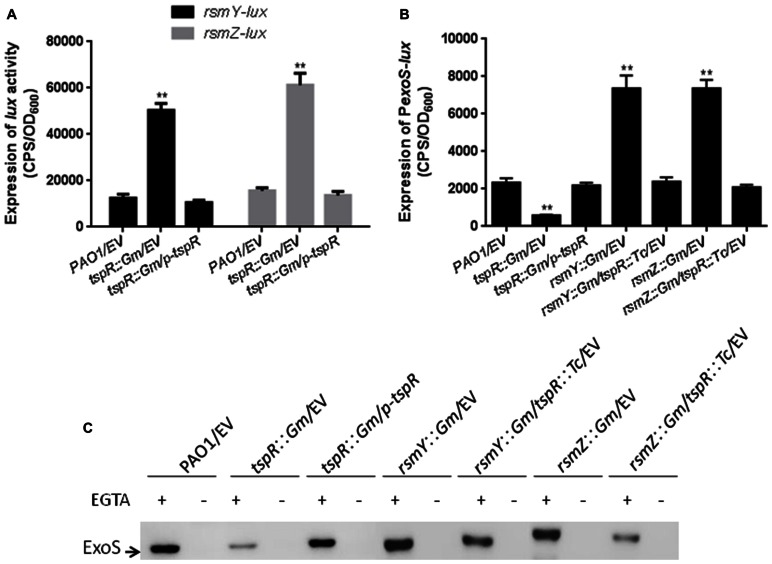
**Two small RNAs, RsmY and RsmZ, are involved in *tspR*-mediated regulation.** **(A)** The expression of *rsmY-lux* and *rsmZ-lux* was strongly induced in the *tspR*::Gm strain compared to that in the wild-type strain. ^∗∗^*P* < 0.005 compared to the wild-type by Student’s *t*-test. **(B)** Mutantion of *tspR* in the *rsmY*::Gm or *rsmZ*::Gm strain reduced the levels of *exoS*. Results shown are the average of triplicate experiments. Error bars indicate SD. **(C)** The roles of RsmY and RsmZ in *tspR*-mediated T3SS gene expression. The indicated strains were grown in LB with or without EGTA to an OD_600_ = 0.6. Whole-cell extracts from the designated strains were separated by SDS-PAGE and subjected to subsequent Western-blotting. EV represents empty vector.

As aforementioned, RetS is a positive regulator of *tspR* expression (**Figure 4**). Previous studies have shown that the RetS-RsmA pathway mainly regulates T3SS and type VI secretion system (T6SS) genes by modulating the levels of two small RNAs RsmY and RsmZ (Goodman et al., 2004; Ventre et al., 2006), this made us wonder if TspR may play a role in the transcription of *rsmY* and *rsmZ*. To address this hypothesis, two promoter-*lux* fusions, *rsmY-lux* and *rsmZ-lux*, were constructed, and their activities were measured in the *tspR*::Gm and the wild-type strains. As shown in **Figure 5A**, *rsmY* and *rsmZ* promoter activities were much higher in the *tspR*::Gm strain than in the wild-type strain, indicating that TspR is a negative regulator of these two sRNAs.

Since *tspR* regulates RsmY/Z, which controls T3SS genes expression, we sought to determine whether *tspR* regulates the expression of T3SS genes through RsmY or RsmZ. To this end, two single mutants (*rsmY*::Gm and *rsmZ*::Gm) and two double mutants (*rsmY*::Gm/*tspR*::Tc and *rsmZ*::Gm/*tspR*::Tc) were constructed. *exoS* promoter activity was evaluated in seven strains, including the wild-type, *tspR*::Gm, and *tspR*::Gm complemented with p-*tspR*, *rsmY*::Gm, *rsmZ*::Gm, *rsmY*::Gm/*tspR*::Tc and *rsmZ*::Gm/*tspR*::Tc strains. As expected, the level of *exoS-lux* was much higher in the *rsmY*::Gm and *rsmZ*::Gm strains, but was much lower in the *tspR*::Gm strain (**Figure 5B**). Interestingly, mutation of *rsmY* or *rsmZ* in the *tspR*::Gm background restored the expression of *exoS* to wild-type levels. We confirmed the above observations by Western-blot analysis with anti-ExoS antibody in the indicated strains (**Figure 5C**). Taken together, these results suggest that these two sRNAs, RsmY and RsmZ, play a role in pathway by which *tspR* regulates the T3SS.

### RetS-RsmY/Z is Involved in the *tspR*-Mediated Regulation of Biofilm Formation

**FIGURE 6:**
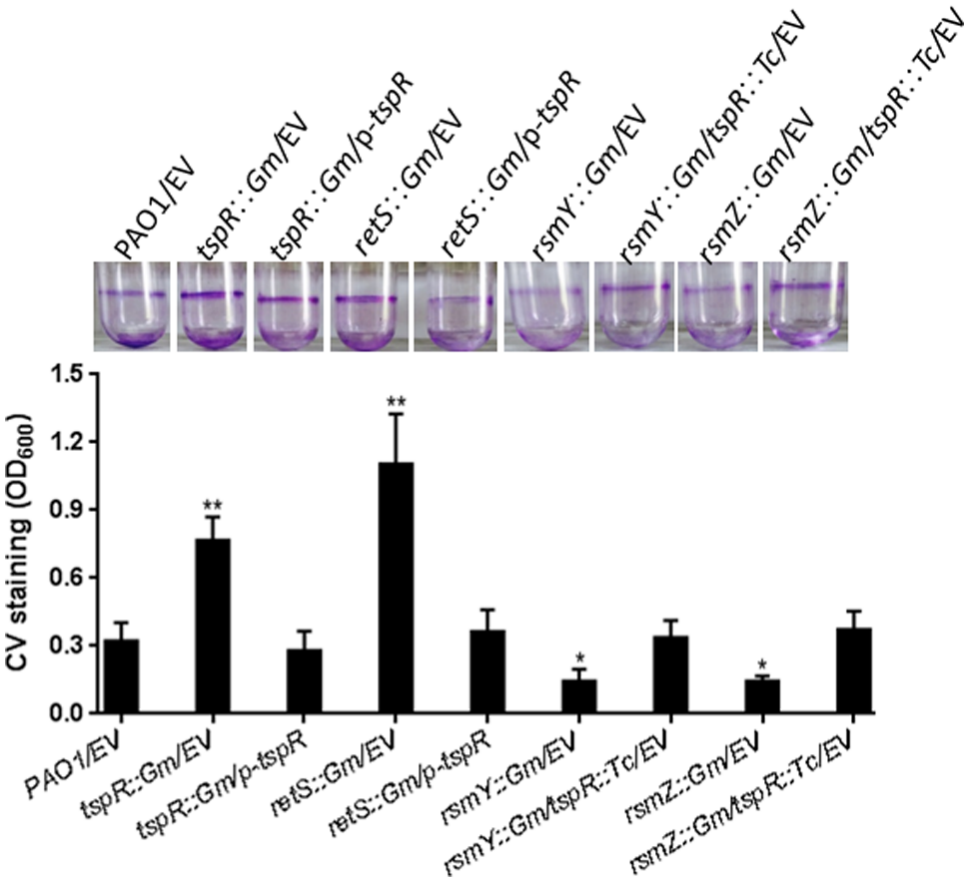
***TspR* regulates biofilm formation through the RetS-RsmY/Z pathway.** Quantification of CV staining of biofilms grown in microtiter plates for 14 h. ^∗^*P* < 0.01 and ^∗∗^*P* < 0.005 compared to the wild-type strain based on Student’s *t*-test. Photos of the tubes binding assay of the indicated strains were taken. EV, empty vector.

The RetS-GacS/A-RsmY/Z-RsmA regulatory pathway reciprocally regulates the expression of virulence factors associated with acute and chronic infections, including the T3SS, the T6SS, and biofilm formation. Overexpression of RsmY/Z as well as deletion of *retS* or *rsmA* in the wild-type strain results in hyperbiofilm formation (Brencic et al., 2009). The decreased expression of *tspR* observed in the *retS*::Gm strain led us to examine whether hyperbiofilm formation of the *retS*::Gm strain is dependent on *tspR* expression. We found that the increased biofilm formation of the *retS*::Gm strain could be restored to wild-type levels by overexpression *tspR*. In contrast, *tspR* negatively regulates RsmY and RsmZ (**Figure 5**). Our data further showed that mutation of RsmY or RsmZ in the *tspR*::Gm strain abolished the hyperbiofilm phenotype (**Figure 6**). Taken together, these results suggest that RetS-RsmY/Z is involved in *tspR*-mediated regulation of biofilm formation.

## Discussion

In the past few years, remarkable progress has been made in our understanding of the structure and function of the T3SS in *P. aeruginosa* and other pathogenic bacteria. However, the networks regulating T3SS are not yet well-understood. In the present study, we constructed a transposon mutagenesis library and identified 15 genes that are associated with T3SS expression. As expected, mutation of *exsA*, *pscC*, or *pcrD* resulted in reduced expression of the T3SS genes (Yahr et al., 1997). We also found that NuoL and ShaC are required for T3SS activity. These proteins are involved in the metabolism of amino acids, energy and nucleotides, which are consistent with previous reports showing that metabolic stresses inhibit the T3SS expression (Linares et al., 2010; Diaz et al., 2011). Recently, an ATP-dependent RNA helicase (*deaD*) was shown to be involved in T3SS expression through the control of ExsA translation (Intile et al., 2015). Interestingly, ClpS, an ATP-dependent protease adaptor, negatively regulates the expression of T3SS. ClpS is known to recognize and bind to specific substrates, and then delivers them to the ClpAP complex for degradation (Erbse et al., 2006; Roman-Hernandez et al., 2011). Thus, we hypothesize that the ClpS-ClpAP system regulates T3SS by modulating the stability of key T3SS transcriptional regulators such as ExsA, GacA, or Vfr. However, a complete understanding of the detailed functions and regulatory mechanisms of these require further investigation. We also observed that the *fimV* mutant was unable to induce the expression of T3SS genes under type III inducing condition. Given that FimV facilitates the secretion of T2SS substrates on solid medium (Michel et al., 2011) and positively regulates the levels of intracellular cAMP (Fulcher et al., 2010), cAMP might play a role in FimV-mediated T3SS expression. However, a group of genes that have been reported to control T3SS were not identified in our current study. There are at least two possible explanations for the omission of these genes in this transposon mutagenesis library. First is the non-random nature of Tn5 insertion, as some regions are preferred for the Tn5 insertion (Jacobs et al., 2003). Second, is that we screened 8,000 Tn insertion mutants, which just barely covers the whole *P. aeruginosa* genome.

We are interested in determining the roles of PA4857 (*tspR*), which encodes a hypothetical protein with an unknown function. On the chromosome, *tspR* is located next to *retS*, which encodes a hybrid kinase RetS that is required for T3SS expression and biofilm formation (Laskowski et al., 2004). TspR is homologous to the multiple antibiotic resistance protein MarC, which is found in a wide variety of bacterial species. Deletion of *tspR* reduced T3SS activity and swarming motility, and promoted biofilm formation, indicating that *tspR* may play a central role in regulating the transition between acute and chronic infection. Moreover, our data also provided insights into the importance of *tspR* in the cytotoxicity of *P. aeruginosa* (**Figures 2A,B**). We also examined the role of *tspR* in virulence by using a mouse pulmonary infection model. Within 48 h, mice inoculated with the *tspR*::Gm strain had a significantly lower bacterial load than mice infected with the wild-type strain (**Figure 2C**). This loss of pathogenicity in the *tspR*::Gm strain confirmed the reduced activity of the T3SS genes (**Figure 1**).

Expression of the T3SS genes is directly controlled by *exsA* (Hovey and Frank, 1995), which is the last gene of the *exsCEBA* operon which is itself strictly regulated by ExsA itself (Yahr and Frank, 1994). The expression of *exsA* is driven by a promoter upstream of *exsC*. In addition, the intergenic region between *exsB* and *exsA* also displayed a weak promoter activity, indicating that it may include a second promoter. This is supported by the identification of a potential transcriptional start site in front of *exsA* (Wurtzel et al., 2012). We also verified this observation in our previous study by showing that VqsM directly binds to and regulates the promoter upstream of *exsA* (Liang et al., 2014). To determine the impact of *tspR* on *exsA* expression, we constructed an *exsA* promoter-*lux* fusion (P*exsC-lux*, **Supplementary Table S1**), and showed that the expression of P*exsC-lux* was lower in the *tspR*::Gm strain than in the wild-type strain (**Figure 3A**), which suggests that TspR is required for transcription of the *exsCEBA* operon. Moreover, we found that *tspR* controls the expression of ExsA at the post-transcriptional level (**Figure 3C**). These results demonstrated that TspR is involved in ExsA regulation, thus regulates T3SS.

**FIGURE 7:**
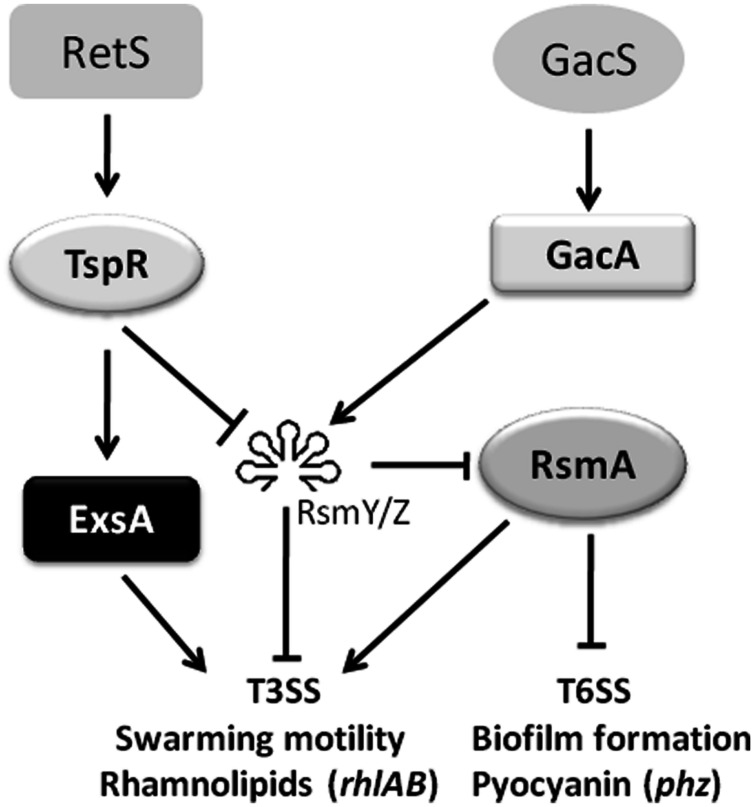
**A schematic diagram showing that *tspR* is involved in the T3SS regulatory network in *P. aeruginosa*.** The potential regulatory pathways and interactions including *tspR* were proposed based on our observations and those of previous studies. RetS and GacS-GacA are two major systems involved in T3SS regulation. In the present study, we identified a modulator, TspR, which tunes T3SS expression through two small sRNAs (RsmY and RsmZ) or the master regulator ExsA. In addition, the activity of *tspR* was shown to be controlled by RetS (blue lines). The solid arrows indicate positive regulation and the solid T-bars present negative regulation.

In addition to modulating the translation of ExsA, *tspR* also regulates two small RNAs RsmY and RsmZ (**Figures 5A,B**). As expected, deletion of *rsmY* or *rsmZ* in the *tspR*::Gm strain restored the activity of *exoS-lux* to wild-type levels (**Figure 5C**), indicating that these two sRNAs participate in *tspR-*mediated T3SS regulation. Moreover, our data showed that RetS, a regulator of exopolysaccharide and T3SS, also positively controls the expression of *tspR* (**Figure 4**). Based on the results of previous studies and our data, we proposed that RetS positively regulates *tspR* expression, and TspR controls T3SS through the sRNAs, RsmY, and RsmZ or the master regulator ExsA (**Figure 7**).

The hallmark differences between acute and chronic *P. aeruginosa* infection are the expression of the T3SS genes and the genes associated with biofilm formation (Goodman et al., 2004). In the present study, TspR regulated both T3SS and biofilm formation. Further studies will be focused on the roles of *tspR* in the transition between acute and chronic infections as well as its genome-wide functions. The findings of this study improved our understanding of the complicated network underlying the regulation of T3SS, and may aid in the design of novel therapeutic strategies that interfere with the expression of T3SS genes and control the pathogenicity of *P. aeruginosa*.

## Author Contributions

MZ and HL conceived and designed the experiments. MZ, JZ, HK, and WK performed the experiments. MZ and HL analyzed the data. MZ and HL wrote the paper.

## Conflict of Interest Statement

The authors declare that the research was conducted in the absence of any commercial or financial relationships that could be construed as a potential conflict of interest.
