# Corrigendum: Modulation of Type III Secretion System in *Pseudomonas aeruginosa*: Involvement of the PA4857 Gene Product

**DOI:** 10.3389/fmicb.2016.00881

**Published:** 2016-06-07

**Authors:** Miao Zhu, Jingru Zhao, Huaping Kang, Weina Kong, Yuanyu Zhao, Min Wu, Haihua Liang

**Affiliations:** ^1^Key Laboratory of Resources Biology and Biotechnology in Western China, Ministry of Education, Department of Life Science, Northwest UniversityXi'an, China; ^2^Department of Basic Science, School of Medicine and Health Science, University of North DakotaGrand Forks, ND, USA

**Keywords:** type III secretion system, *tspR*, bacterial virulence, regulatory mechanisms, *Pseudomonas aeruginosa*

Due to an oversight, in the original article Min Wu and Yuanyu Zhao were not included in the author list. They have now been added and the correct author list can be found above. We have also updated the Acknowledgements section to reflect the financial support received by these two authors.

The correction does not affect the scientific validity of the results.

The original article has been updated.

## Conflict of interest statement

The authors declare that the research was conducted in the absence of any commercial or financial relationships that could be construed as a potential conflict of interest.

