# Supplementary material for: Modulation of Type III Secretion System in Pseudomonas aeruginosa: Involvement of the PA4857 Gene Product
Source: Front Microbiol. 2016 Jan 28;7:7. doi: 10.3389/fmicb.2016.00007 (PMC4729953; doi:10.3389/fmicb.2016.00007)
Supplement: Supplementary file 1 [file Table_1.PDF]

Table S1. Bacterial strains and plasmids used in this study

Strain or plasmid	Relevant characteristics	Source
E. coli		
DH5α	*F⁻ φ80lacZ ΔM15 Δ(lacZYA-argF)U169 recA1 endA1 hsdR17(rk⁻, mk⁺)phoA supE44 thi-1 gyrA96 relA1 tonA*	Stratagene
P. aeruginosa		
PAO1	Wild type	This lab
tspR::Tn	PA4857 transposon mutagenesis mutant	This study
tspR::Gm	*tspR* (*PA4857*) insertion mutant of PAO1; Gm^r	This study
tspR::Tc	*tspR* (*PA4857*) insertion mutant of PAO1; Tc^r	This study
retS::Gm	*retS* insertion mutant of PAO1; Gm^r	This study
exsA::Gm	*exsA* insertion mutant of PAO1; Gm^r	This study
rsmY::Gm	*rsmY* insertion mutant of PAO1; Gm^r	This study
rsmZ::Gm	*rsmZ* insertion mutant of PAO1; Gm^r	This study
rsmY::Gm/*tspR*::Tc	*tspR* and *rsmY* double mutant of PAO1; Gm^r, Tc^r	This study
rsmZ::Gm/*tspR*::Tc	*tspR* and *rsmZ* double mutant of PAO1; Gm^r, Tc^r	This study
tspR::Gm*/CTX-tspR*	*tspR*::Gm complemented strain, derived from *tspR*::Gm and Mini-CTX-*tspR*; Gm^r, Tc^r	This study
tspR::Gm */p-tspR*	*tspR*::Gm complemented strain, derived from *tspR*::Gm and pAK1900-*tspR*; Gm^r, Cb^r	This study
retS::Gm*/p-retS*	*retS*::Gm complemented strain, derived from *retS*::Gm and pAK1900-*retS*; Gm^r, Cb^r	This study
retS::Gm*/p-tspR*	*retS*::Gm strain carrying plasmid pAK1900-*tspR*; Gm^r, Cb^r	This study
**Plasmids**		
pBT20	Mini-Tn*M* delivery vector, Ap^r, Gm^r	(Kulasekara, *et al.*, 2005)
pEX18Ap	*oriT*⁺ *sacB*⁺ gene replacement vector with multiple-cloning site from pUC18; Ap^r	(Hoang, *et al.*, 1998)
pEX18Tc	*oriT*⁺ *sacB*⁺ gene replacement vector with multiple-cloning site from pUC18; Tc^r	(Hoang, *et al.*, 1998)
pPS858	Source plasmid of Gm^r cassette; Gm^r, Ap^r	(Hoang, *et al.*, 1998)
pRK2013	Broad-host-range helper vector; Tra^+, Kn^r	(Ditta, *et al.*, 1980)
pMS402	Expression reporter plasmid carrying the promoterless *luxCDABE* gene; Kn^r, Tmp^r	(Duan, *et al.*, 2003)
pAK1900	*E. coli-P. aeruginosa* shuttle cloning vector carrying p*lac* upstream of MCS; Ap^r, Cb^r	(Poole, *et al.*, 1993)
exsA-FLAG-A	*exsA* genes with P*tac* and FLAG	(Li, *et al.*, 2013)
exsA-FLAG-S	*exsA* genes with P*tac*, promoter region and FLAG	(Li, *et al.*, 2013)

mini-CTX-*lacZ*	Integration plasmid; Tc^{r}	(Becher & Schweizer, 2000)
mini-CTX-*lux*	Integration plasmid; Tc^{r}	(Becher & Schweizer, 2000)
pEX-*tspR*$_{Gm}$	*tspR* deletion plasmid, pEX18Tc with 1959 bp upstream region, Gm^{r} cassette from pPS858 and 1463 bp downstream region of *tspR*; Tc^{r}, Gm^{r}	This study
pEX-*tspR*$_{Tc}$	*tspR* deletion plasmid, pEX18Ap with 1959 bp upstream region, Tc^{r} cassette from mini-CTX-lacZ and 1463 bp downstream region of *tspR*; Tc^{r}	This study
pEX-*retS*	*retS* deletion plasmid, pEX18Ap with 1958 bp upstream region, Gm^{r} cassette from pPS858 and 1981 bp downstream of *retS*; Ap^{r}, Gm^{r}	This study
pEX-*exsA*	*exsA* deletion plasmid, pEX18Ap with 1963 bp upstream region, Gm^{r} cassette from pPS858 and 1972 bp downstream region of *exsA*; Ap^{r}, Gm^{r}	This study
pEX-*rsmY*	*rsmY* deletion plasmid, pEX18Ap with 1963 bp upstream region, Tc^{r} cassette from mini-CTX-lacZ and 1979 bp downstream of *rsmY*; Ap^{r}, Tc^{r}	This study
pEX-*rsmZ*	*rsmZ* deletion plasmid, pEX18Ap with 1974 bp upstream region, Tc^{r} cassette from mini-CTX-lacZ and 1972 bp downstream of *rsmZ*; Ap^{r}, Gm^{r}	This study
p-*tspR*	pAK1900 with the entire *tspR* gene; Ap^{r}	This study
p-*retS*	pAK1900 with the entire *retS* gene; Ap^{r}	This study
p*exoS-lux*	pMS402 containing *exoS* promoter region; Kn^{r}, Tmp^{r}	(Duan, *et al.*, 2003)
P*exoY-lux*	pMS402 containing *exoY* promoter region; Kn^{r}, Tmp^{r}	(Duan, *et al.*, 2003)
P*exoT-lux*	pMS402 containing *exoT* promoter region; Kn^{r}, Tmp^{r}	(Duan, *et al.*, 2003)
P*tspR-lux*	pMS402 containing *tspR* promoter region; Kn^{r}, Tmp^{r}. The promoter region of *tspR* is -522 to +77 from *tspR* translational starting site.	This study
P*retS-lux*	pMS402 containing *retS* promoter region; Kn^{r}, Tmp^{r}. The promoter region of *retS* is -378 to +139 from *retS* translational starting site.	This study
P*exsC-lux*	pMS402 containing *exsC* promoter region; Kn^{r}, Tmp^{r}	(Kong, *et al.*, 2013)
CTX-*tspR-lux*	Integration plasmid, CTX6.1 with a fragment of pKD-*tspR*; Kn^{r}, Tc^{r}	This study
CTX-*exoS-lux*	Integration plasmid, CTX6.1 with a fragment of pKD-*exoS*; Kn^{r}, Tc^{r}	(Liang, *et al.*, 2011)

CTX-*exoT-lux*	Integration plasmid, CTX6.1 with a fragment of pKD-*exoT*; Kn^r, Tc^r	(Liang, *et al.*, 2011)
CTX-*exoY-lux*	Integration plasmid, CTX6.1 with a fragment of pKD-*exoY*; Kn^r, Tc^r	(Liang, *et al.*, 2011)

Reference

Becher A & Schweizer HP (2000) Integration-proficient *Pseudomonas aeruginosa* vectors for isolation of single-copy chromosomal *lacZ* and *lux* gene fusions. *BioTechniques* **29**: 948-953.

Ditta G, Stanfield S, Corbin D & Helinski DR (1980) Broad host range DNA cloning system for gram-negative bacteria: construction of a gene bank of *Rhizobium meliloti*. *Proc Natl Acad Sci U S A* **77**: 7347-7351.

Duan K, Dammel C, Stein J, Rabin H & Surette MG (2003) Modulation of *Pseudomonas aeruginosa* gene expression by host microflora through interspecies communication. *Mol Microbiol* **50**: 1477-1491.

Hoang TT, Karkhoff-Schweizer RR, Kutchma AJ & Schweizer HP (1998) A broad-host-range Flp-FRT recombination system for site-specific excision of chromosomally-located DNA sequences: application for isolation of unmarked *Pseudomonas aeruginosa* mutants. *Gene* **212**: 77-86.

Kong W, Chen L, Zhao J, Shen T, Surette MG, Shen L & Duan K (2013) Hybrid sensor kinase PA1611 in *Pseudomonas aeruginosa* regulates transitions between acute and chronic infection through direct interaction with RetS. *Mol Microbiol* **88**: 784-797.

Kulasekara HD, Ventre I, Kulasekara BR, Lazdunski A, Filloux A & Lory S (2005) A novel two-component system controls the expression of *Pseudomonas aeruginosa* fimbrial cup genes. *Mol Microbiol* **55**: 368-380.

Li K, Xu C, Jin Y, *et al.* (2013) SuhB is a regulator of multiple virulence genes and essential for pathogenesis of *Pseudomonas aeruginosa*. *MBio* **4**: e00419-00413.

Liang H, Duan J, Sibley CD, Surette MG & Duan K (2011) Identification of mutants with altered phenazine production in *Pseudomonas aeruginosa*. *J Med Microbiol* **60**: 22-34.

Poole K, Neshat S, Krebes K & Heinrichs DE (1993) Cloning and nucleotide sequence analysis of the ferripyoverdine receptor gene *fpvA* of *Pseudomonas aeruginosa*. *J Bacteriol* **175**: 4597-4604.
