# Supplementary material for: Modulation of Type III Secretion System in Pseudomonas aeruginosa: Involvement of the PA4857 Gene Product
Source: Front Microbiol. 2016 Jan 28;7:7. doi: 10.3389/fmicb.2016.00007 (PMC4729953; doi:10.3389/fmicb.2016.00007)
Supplement: Supplementary file 2 [file Table_2.PDF]

Table S2. Primers used in this study

Primer	Sequence (5'→3')[a]	Application
pEX-*tspR*-up-S	TGAgaattcACTACGTGCAGACCATCC	Constructing *tspR* mutant
pEX-*tspR*-up-A	TAGtctagAGGTAGACGCTGAACAGC	
pEX-*tspR*-down-S	TAGtctagaGCGCAGATCATCGTCACC	
pEX-*tspR*-down-A	TCAaagctTGTGGATGCCGACGTAGG	
pEX-*exsA*-up-S	TGTgaattcATCGACGCGAAGATGCAG	Constructing *exsA* mutant
pEX-*exsA*-up-A	TAGtctagaTCTGCTTTCGGCCAAGAG	
pEX-*exsA*-down-S	TATtctagaGGAAGGACGAATGCCGGG	
pEX-*exsA*-down-A	TGTaagcttGCATAGCGCAGCGGGAAG	
pEX-*retS*-up-S	TAGggtaccAAGGCCGAGGACCTGAAG	Constructing *retS* mutant
pEX-*retS*-up-A	TAGtctagaACCGAGCAGAAGCAGCAG	
pEX-*retS*-down-S	TGAtctagaGTGGAGCTGTCGCAATTG	
pEX-*retS*-down-A	TGAaagcttAGACGTTCGGCGAGAGTT	
pEX-*rsmY*-up-A	TAAtctagaGATTACGCATCTCTGCG	
pEX-*rsmY*-down-S	TAAtctagaGCGTCGCTCAGACCACGC	
pEX-*rsmY*-down-A	TATaagcttATCCAGCGCATCAGCCAT	
pEX-*rsmZ*-up-S	TGAgaattcCCTATACCATCCAGGTCG	Constructing *rsmZ* mutant
pEX-*rsmZ*-up-A	TAGtctagaGTTGCGTGTTCCCTGTAC	
pEX-*rsmZ*-down-S	TTAtctagaGCGGGGTAATACCCCGCC	
pEX-*rsmZ*-down-A	TATaagcttAACGCCCGCCCTCGCGAC	
Com-*tspR*-S	TATggatCCTACTGGGTCGGCGAAC	Constructing p-*tspR* plasmid
Com-*tspR*-A	TATaagcttGCTCAGCCTGGGAACAGC	
Com-*retS*-S	TATggtaccGGCGACTACGCCAAGGGC	Constructing p-*retS* plasmid
Com-*retS*-A	TATaagcttTACGGGAGCCGGGACCAC	
tspR-lux-S	TGActcgagTGAGCTGATCGCCTACTG	Constructing *tspR-lux* plasmid
tspR-lux-A	TGCggatccAGGCTGATGAAGCAGGAC	
retS-lux-S	TGActcgagGCTATCCGGGCGACTACG	Constructing *retS-lux* plasmid
retS-lux-A	TAAggatccGGTTGGCGCTGGGAGTAG	
Tc-S	TTTtctagaCGGTCGCTACCATTACCAGT	Amplifying Tc^r cassette
Tc-A	TTTtctagaCTGGTGAGTCAAGGGTTGGT	

[a] lowercases represent the Restriction sites;
